# The H2B ubiquitin-protein ligase RNF40 is required for somatic cell reprogramming

**DOI:** 10.1038/s41419-020-2482-4

**Published:** 2020-04-27

**Authors:** Wanhua Xie, Michaela Miehe, Sandra Laufer, Steven A. Johnsen

**Affiliations:** 1 The Precise Medicine Center, Shenyang Medical College, Shenyang, China; 2 Department of Tumor Biology, University Medical Center Hamburg-Eppendorf, Hamburg, Germany; 3 Department of General, Visceral and Pediatric Surgery, University Medical Center Gottingen, Gottingen, Germany; 4 Institute of Experimental Pharmacology and Toxicology, University Medical Center Hamburg-Eppendorf, Hamburg, Germany; 5 Gene Regulatory Mechanisms and Molecular Epigenetics Lab, Division of Gastroenterology and Hepatology, Mayo Clinic, Rochester, MN USA

**Keywords:** Histone post-translational modifications, Induced pluripotent stem cells

## Abstract

Direct reprogramming of somatic cells to induced pluripotent stem cells (iPSCs) requires a resetting of the epigenome in order to facilitate a cell fate transition. Previous studies have shown that epigenetic modifying enzymes play a central role in controlling induced pluripotency and the generation of iPSC. Here we show that RNF40, a histone H2B lysine 120 E3 ubiquitin-protein ligase, is specifically required for early reprogramming during induced pluripotency. Loss of RNF40-mediated H2B monoubiquitination (H2Bub1) impaired early gene activation in reprogramming. We further show that RNF40 contributes to tissue-specific gene suppression via indirect effects by controlling the expression of the polycomb repressive complex-2 histone methyltransferase component EZH2, as well as through more direct effects by promoting the resolution of H3K4me3/H3K27me3 bivalency on H2Bub1-occupied pluripotency genes. Thus, we identify RNF40 as a central epigenetic mediator of cell state transition with distinct functions in resetting somatic cell state to pluripotency.

## Introduction

Somatic cells can be directly reprogrammed into a pluripotent state by forced expression of the Yamanaka factors Oct4, Sox2, Klf4, and c-Myc^1^. Even though these factors can be stably activated in somatic cells, the generation of high-quality induced pluripotent stem cells (iPSCs) is highly inefficient, with low frequency and a long latency^2,3^. Therefore, a better understanding of the mechanisms driving somatic cell reprogramming will be crucial for maximizing potential clinical application of this technology.

Epigenetic modifications, including DNA methylation and post-translational histone modifications, have been demonstrated to activate or repress particular important subsets of genes during cell fate transition. In the last decades, some key histone modifying enzymes, and by extension the resulting histone modifications themselves have been demonstrated to play important roles in resetting somatic cells and enabling a transition to pluripotency. Changes in H3K9me2/3, H3K79me2/3, H3K4me3, and H3K27me3 were suggested to influence the reprogramming efficiency^4–6^. For example, the H3K79 methyltransferase DOT1L, H3K4 methyltransferase MLL1, and H3K27 demethylase UTX have all been identified as epigenetic barriers to iPSC generation^5,7^, while the Polycomb Repressor Complex members EZH2, EED, and SUZ12, which catalyze the methylation of H3K27, were required for efficient reprogramming^5^. The monoubiquitination of histone H2B is unique in comparison to other histone modifications in that it represents the conjugation of a 76-amino acid ubiquitin moiety to lysine 120 of H2B (H2Bub1), thereby imposing significant steric effects on chromatin structure^8,9^. Importantly, we and others demonstrated that the two members of the obligate heterodimeric ubiquitin ligase complex, RNF20 and RNF40, play an essential role in directing cell identity and are required for the stimulation of lineage-specific genes during stem cell differentiation^10–13^. However, while a central role for H2Bub1 in cell differentiation has been established, its influence on epigenetic reprogramming during induced pluripotency has not been clearly defined.

During epigenetic reprogramming, somatic cells go through various cell fate transitions resulting from a cascade of transcriptional events, which is accompanied by increased cell proliferation^14,15^. This transcriptional event is suggested to be an important bottleneck during reprogramming^16^. A number of factors controlling cell cycle have been shown to influence somatic cell reprogramming. Notably, inhibition of the p53-p21 pathway increases reprogramming efficiency by removing the barrier to cell cycle progression^17,18^. Similarly, one of the central Yamanaka factors, c-Myc, is an established oncogene which, like it functions in cancer, enhances the transcription of cell cycle-related genes during reprogramming to increase iPSC generation^19,20^. An influence of H2Bub1 on cell proliferation has been observed in various cell types, although the results have varied with some reports suggesting a pro-proliferative function and others reporting an anti-proliferative role of RNF20 and/or RNF40 in controlling proliferation^21–28^. Furthermore, H2Bub1 was reported to be enriched at centromeric regions during the G2/M phase, and loss of RNF20/40 was suggested to affect chromosome segregation and result in cell cycle arrest^29,30^.

Epigenetic profiling studies have revealed that the promoters of many lineage-specific genes in somatic cells and pluripotent stem cells simultaneously exhibit both activating (H3K4me3) and repressive (H3K27me3) modifications in an epigenetic pattern termed “bivalence”^31,32^. This state is thought to place lineage-specific genes in a “poised” state which enables their rapid activation following specific stimuli during cell fate transitions^32^. During somatic cell reprogramming, transcriptional silencing of fibroblast-specific genes and activation of pluripotency genes are executed by the establishment or resolution of epigenetic bivalency, respectively^14,33^. Our previous studies revealed that the H2B ubiquitin ligase RNF40 directs lineage-specific gene transcription by controlling H3K4me3 and H3K27me3^11,12^. However, the role of H2Bub1 in governing epigenetic bivalency and induced pluripotency during somatic cell reprogramming remained unclear.

In this study, we show for the first time that perturbation of H2B monoubiquitination via loss of its E3 ubiquitin ligase RNF40 impairs iPSC generation. Consistent with previous studies, deletion of *Rnf40* inhibits cell proliferation and induces cell cycle arrest, consistent with the importance of cell cycle progression during somatic cell reprogramming. In addition, *Rnf40* deletion affects the silencing of cell lineage-specific genes and activation of pluripotency genes by controlling the bivalent histone marks H3K4me3 and H3K27me3 during somatic cell reprogramming. Together those results uncover a previously unknown function of H2Bub1 and RNF40 in cellular reprogramming via regulating cell cycle genes and epigenetic bivalency.

## Materials and methods

### MEF generation and reprogramming

The generation of MEFs from the conditional *Rnf40* knockout mouse strain containing the global, constitutively expressed tamoxifen-inducible Cre recombinase (Rosa26-CreERT2) was described previously^12^. All animal studies were performed in compliance with the requirements of the German Animal Welfare Act and were approved by the institutional animal care and use committee at the University Hospital Hamburg-Eppendorf (approval number ORG 673). Primary MEFs were grown in high-glucose GlutaMAX™-DMEM (Invitrogen) supplemented with 10% FBS Superior (Biochrom), 1% penicillin–streptomycin (Sigma-Aldrich), and 1% non-essential amino acids (Invitrogen) at 37 °C with 5% CO_2_. MEFs were cultured in growth medium containing 250 nM of 4-hydroxytamoxifen (4-OHT) for 5 days to induce Cre-mediated *Rnf40* deletion. For iPSC generation, early passage MEFs (≤3 passages) were plated in equal numbers onto 6-well plates and subsequently infected with two doses of retrovirus carrying Oct4, Sox2, Klf4, and c-Myc^1^. The viral supernatant was removed 48 h after infection and transduced MEFs were seeded in equal numbers onto mitomycin-C-treated feeder MEFs or feeder-free plates and defined as day 0 post-transduction. Subsequently, pluripotency was induced with feeder cell co-culture in ESC medium (DMEM with 15% FBS, non-essential amino acid, L-glutamine, sodium pyruvate, β-mercaptoethanol, penicillin/streptomycin and 1000 U ml^−1^ leukemia inhibitory factor), or in feeder-free N2B27 2i/LIF medium (KO-DMEM with 15–20% knockout serum replacement, B27 supplement, N2 supplement, glutamine, non-essential amino acids, β-mercaptoethanol, penicillin–streptomycin, BSA, and leukemia inhibitory factor). Cell proliferation was measured as confluence using a Celigo® S imaging cytometer (Nexcelom Bioscience LLC).

### Western blot and gene expression analysis

Western blot was performed essentially as described previously^22^. Briefly, cells were lysed in radioimmunoprecipitation buffer (PBS with 1% Nonidet P40, 0.5% sodium deoxycholate, and 0.1% SDS), solubilized by brief sonication, and protein extracts were incubated with Laemmli SDS loading dye at 95 °C for 10 min. Equal amounts of samples were separated by PAGE and analyzed by western blot analysis using the indicated antibodies (listed in Supplementary Table 1).

For gene expression analysis, total RNA was extracted using QIAzol reagent (Qiagen) according to the manufacturer’s instructions. One microgram of RNA from each sample was used to synthesize the first strand cDNA. Gene expression was verified and detected by quantitative real-time PCR as described before^22^ using the primers listed in Supplementary Table 2. Gene expression levels were further normalized to the housekeeping gene *36B4*. All experiments were performed in biological triplicate with technical duplicates.

### Cell staining

For immunofluorescent staining, reprogramming was carried out on coverslips coated with 0.1% gelatin. Antibodies against SSEA1 (MAB2155; R&D systems) and Nanog (AF1997; R&D) were applied at a dilution of 1:500. Alkaline phosphatase staining was performed using an alkaline phosphatase detection kit (Millipore) according to the manufacturer’s instructions.

### ChIP and ChIP-qPCR

ChIP for RNA Polymerase II was performed as previously described^12^. Cells were crosslinked with 1% formaldehyde for 15 min. Chromatin was fragmented by sonication using a Bioruptor Pico (Diagenode) for 25 cycles with 30 s on and 30 s off. For each precipitation reaction, fragmented chromatin was incubated overnight at 4 °C with 1 μg antibody against RNA Polymerase II (sc-899; Santa Cruz) or non-specific IgG (ab37415; Abcam) as a negative control. Immune complexes were further pulled down with protein A-sepharose at 4 °C for 1 h and immunopercipitated DNA was purified by phenol:chloroform extraction and ethanol precipitation in the presence of linear acrylamide (AM9520; Invitrogen). Input DNA (10% of the amount utilized for immunoprecipitation) was prepared from chromatin extracts and used for the normalization of ChIP samples. Chromatin occupancy was determined by quantitative real-time PCR using the indicated primers (Supplementary Table 2), normalized to input DNA samples and displayed as “% of input”.

### mRNA-sequencing and ChIP-sequencing data analysis

The accession numbers for published datasets utilized in this study are listed in Supplementary Table 3. mRNA-seq data were re-analyzed and mapped to the mouse reference transcriptome (UCSC mm9). Differentially expressed genes and normalized counts of each gene were obtained by carrying out DESeq analysis^34^. Gene set enrichment analysis (GSEA) and gene ontology (GO) were performed to identify significantly enriched gene sets as previously described^35,36^.

ChIP-seq data were mapped to the mouse reference genome (UCSC mm9) using bowtie2^37^. Significantly enriched regions were next identified by performing MACS2 analysis^38^. Each processed bam file was normalized to reads per kilobase per million (RPKM) using the BamCoverage tool in DeepTools^39^ and visualized using the Integrative Genomics Viewer (version 2.3.14)^40^. The table containing mouse transcriptional start sites (TSS) was obtained from the UCSC table browser and used to generate heatmaps^41^. Genes related to cell proliferation, which are activated early in reprogramming, were identified by GSEA and selected to generate a heatmap showing histone modification occupancy. Bivalent promoters were obtained by intersecting the intervals of H3K4me3 and H3K27me3 near TSS regions, and then further classified into H2Bub1-enriched and -unenriched clusters by K-means.

## Results

### Loss of RNF40 and associated H2Bub1 impairs reprogramming of MEFs to pluripotency

**Fig. 1:**
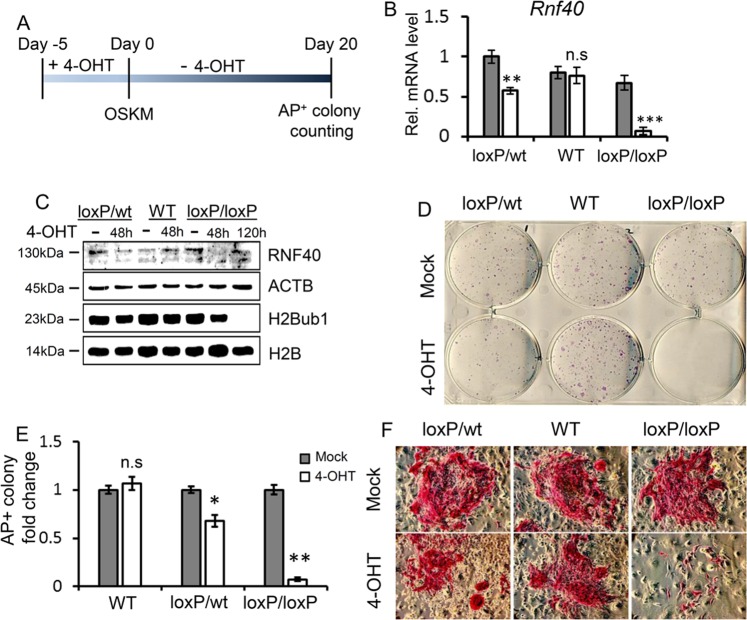
RNF40 is required for somatic epigenetic reprogramming. **a** Timeline of inducing *Rnf40* knockout by the addition of 4-hydroxytamoxifen (4-OHT) and iPSC generation. **b** qRT-PCR analysis of *Rnf40* mRNA levels in *Rnf40* wild-type (*Rnf40*^wt^), heterozygous (*Rnf40*^loxP/wt^), and homozygous (*Rnf40*^loxP/loxP^) MEFs in response to 4-OHT treatment. MEF cultures were supplemented with or without 250 nM of 4-OHT for 5 days (white bars, 4-OHT; gray bars, untreated). Data are shown as “relative mRNA levels”, mean ± SD (*n* = 3). *p < 0.05, **p < 0.001, **p < 0.0001, n.s: *p* > 0.05, calculated with two-tailed unpaired *t*-test. Variance was similar between groups. **c** Western blot shows the protein level of RNF40, H2Bub1, ACTB (loading control), and H2B (loading control) in WT, loxP/wt, and loxP/loxP MEFs with and without 4-OHT treatment. In order to induce *Rnf40* deletion, MEFs were cultured in the presence of 250 nM 4-OHT for 48 or 120 h (only in homozygous MEFs). **d–f** Numbers of AP + iPSC colonies. Alkaline phosphatase (AP) staining was performed 20 days after OSKM transduction of 5000 WT, loxP/wt, and loxP/loxP MEFs with or without adding 4-OHT. Experiments were repeated twice independently. *p < 0.05, **p < 0.001, n.s: *p* > 0.05, calculated with two-tailed unpaired *t*-test.

Previous studies revealed that the H2B ubiquitin ligases RNF20 and RNF40 are essential for human mesenchymal stem cell and mouse embryonic stem cell differentiation by controlling cell lineage-specific gene expression^10,11,13^. Thus, we hypothesized that RNF40-mediated H2B monoubiquitination may also influence somatic epigenetic reprogramming. In order to test this, we utilized mouse embryo fibroblasts containing wild-type, heterozygous (*Rnf40*^wt/loxP^), or homozygous (*Rnf40*^loxP/loxP^) alleles of the *Rnf40* gene^12^ derived from mice expressing the tamoxifen-inducible CreERT2 transgene under the control of the ubiquitously expressed *Rosa26* locus (Supplementary Fig. 1A). MEFs were treated with or without 4-hydroxytamoxifen (4-OHT) as described previously^12^ to delete the *Rnf40* gene (Fig. 1b, c and Supplementary Fig. 1B, C). In order to investigate the effect of *Rnf40* loss on somatic cell reprogramming, MEFs were replated in equal numbers following *Rnf40* deletion and infected with a cocktail of retroviruses carrying the four Yamanaka transcription factors (Oct4, Sox2, Klf4, and Myc)^42^. The efficiency of iPSC generation was analyzed at day 20 by counting the alkaline phosphatase-positive (AP+) colonies (Fig. 1a)^42^. To ensure that reprogramming had occurred, we verified that wild-type-derived iPSC also expressed the stem cell markers SSEA1 and Nanog (Supplementary Fig. 1D). Remarkably, the number of AP+ colonies was significantly reduced in the heterozygous *Rnf40*^wt/loxP^ group and almost completely absent in homozygous *Rnf40*^loxP/loxP^ MEFs after 4-OHT induction, while wild-type cells could be readily induced to pluripotency (Fig. 1d, e). Notably, even the few AP+ *Rnf40*-deficient iPSC colonies that formed were very small (Fig. 1f). Thus, these data suggest that RNF40 is required for somatic cell reprogramming to pluripotency.

### Deletion of *Rnf40* inhibits cell proliferation

**Fig. 2:**
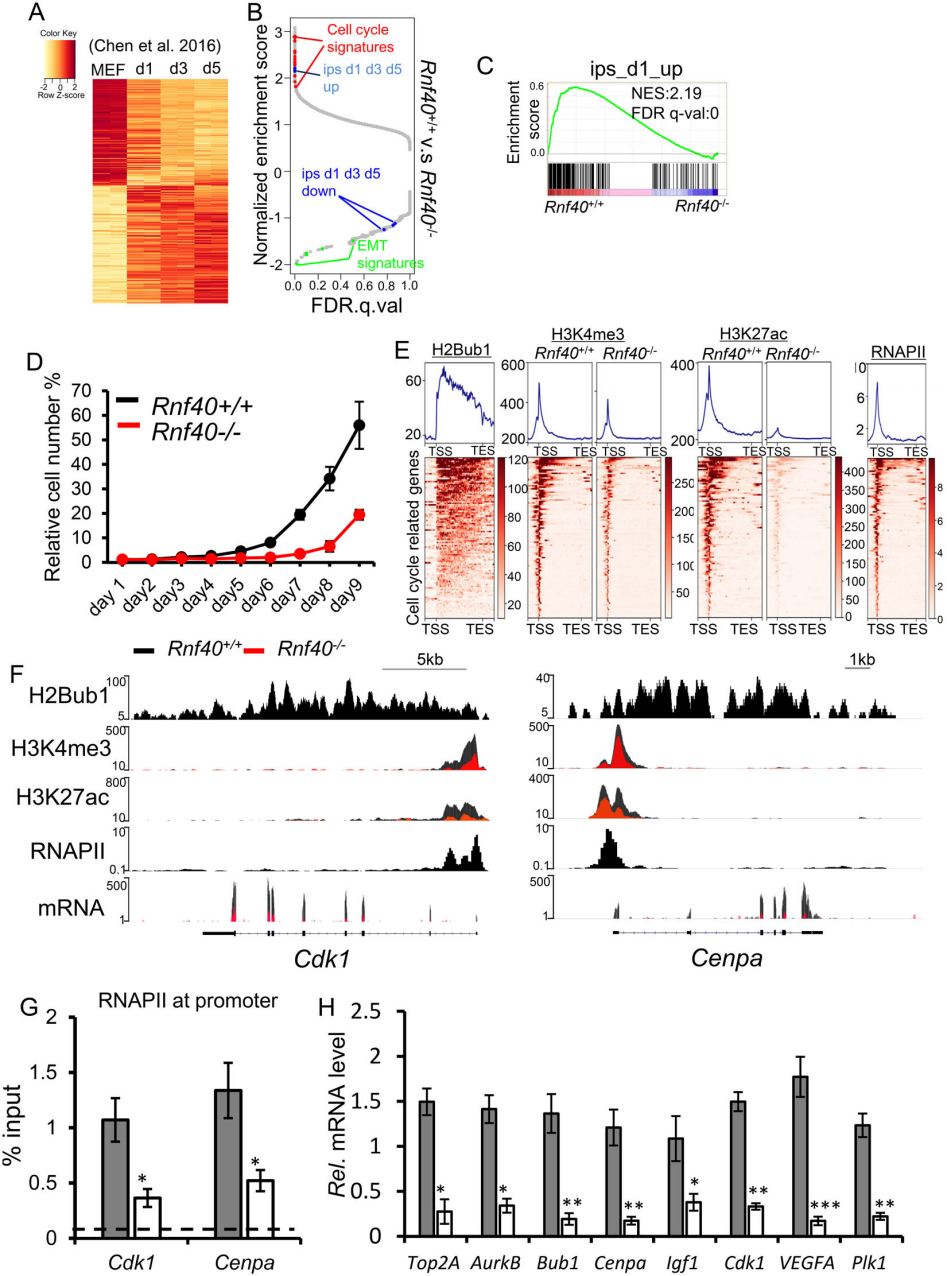
RNF40 controls cell cycle-related gene expression patterns. **a** Heatmap shows the differentially expressed genes following OSKM transduction for 1, 3, or 5 d. The expression array data from GSE67462 were re-analyzed. The differentially regulated genes were selected based on *p* value < 0.05 and |log2 fold change| > 0.3. **b** Gene set enrichment analysis (GSEA) of the RNA-Seq data of *Rnf40*-proficient and -deficient MEFs. FDR.q.val, false discovery rate *q*-value. **c** GSEA on mRNA-seq data demonstrates a significant decrease in the expression of genes activated at day 1 following OSKM transduction in *Rnf40*-deficient MEFs. NES, normalized enrichment score; FDR, false discovery rate. **d** Loss of *Rnf40* impaired cell proliferation. Cellular confluence was measured over time using live cell imaging and shown relative to the control condition. **e** Aggregate profiles (above) and heatmaps (below) show the occupancy of H2Bub1, H3K4me3, H3K27ac, and RNA Polymerase II (RNAPII) on cell cycle-related genes from 2 kb upstream of TSS to 2 kb downstream of TES. Genes are sorted according to H2Bub1 occupancy from high to low. Color key for each heatmap is shown on the right. **f** The profiles show the occupancy of H2Bub1, H3K4me3, H3K27ac, and RNAPII as well as normalized RNA reads on the *Cdk1* (left) and *Cenpa* (right) genes in *Rnf40*+/+ (black) and *Rnf40*−/− (red) MEFs. **g** ChIP-qPCR analyses of RNAPII occupancy at the promoter region of the *Cdk1* and *Cenpa* genes. The dotted line represents the average signal for IgG (negative control). **h** qRT-PCR analysis of the mRNA levels of cell cycle-related genes (*Top2a*, *Aurkb*, *Bub1*, *Cenpa*, *Cdk1*, and *Plk1*) in *Rnf40*+/+ and *Rnf40*−/− MEFs. Data are shown as “relative mRNA levels”, mean ± SD (*n* = 3). *p < 0.05, **p < 0.001, **p < 0.0001, n.s: *p* > 0.05, calculated with two-tailed unpaired t-test.

Previous work has demonstrated that the early phase of reprogramming requires a characteristic increase in cell proliferation and downregulation of genes associated with an epithelial-to-mesenchymal transition (EMT)^15,43^. Differential expression analysis of publically available data followed by GO analysis confirmed this finding at days 1, 3, and 5 after OSKM transduction (Fig. 2a, Supplementary Table 4, and Supplementary Fig. 2A)^14^. Importantly, gene set enrichment analyses (GSEA) revealed that loss of *Rnf40* specifically and differentially affected cell cycle and EMT signatures (Fig. 2b and Supplementary Fig. 2B, C) and displayed a downregulation of genes normally upregulated during reprogramming (Fig. 2b, c). Importantly, the cell cycle-related genes displaying upregulation at the early reprogramming phase (at days 1, 3, and 5) were significantly decreased by *Rnf40* deletion (Supplementary Fig. 2E). Moreover, the EMT-related gene sets showing downregulation at the early reprogramming phase were significantly enriched in *Rnf40*-deleted MEFs (Supplementary Fig. 2F). We further confirmed that deletion of *Rnf40* significantly impaired cell proliferation (Fig. 2d). Thus, these findings support a role for RNF40-mediated H2B monoubiquitination in maintaining cell cycle-regulated gene expression patterns essential for the early stages of somatic cell reprogramming.

We next sought to more thoroughly characterize the role of H2Bub1 in controlling cell cycle-related genes. Consistent with a positive role in maintaining active gene transcription, H2Bub1 was readily present on the gene body of cell cycle-related genes and correlated with active histone marks (H3K4me3 and H3K27ac) and RNA Polymerase II (RNAPII) occupancy (Fig. 2e). Deletion of *Rnf40* resulted in a significant decrease in H3K4me3 occupancy and nearly complete loss of H3K27ac on these genes (Fig. 2e). ChIP-seq profiles for *Cdk1* and *Cenpa* are shown as examples of cell cycle-related genes which display H2Bub1 across the gene body, as well as the presence of the active histone marks H3K4me3 and H3K27ac and RNAPII near the transcriptional start sites (TSS) (Fig. 2f). Notably, loss of H2Bub1 resulted in a significant decrease in H3K4me3 and H3K27ac occupancy (Fig. 2f) and decreased RNAPII occupancy at the *Cdk1* and *Cenpa* promoters (Fig. 2g). Importantly, downregulation of several cell cycle-related genes (*Top2a*, *Aurkb*, *Bub1*, *Cenpa*, *Cdk1*, and *Plk1*) in response to *Rnf40* deletion could be confirmed by quantitative real-time RT-PCR (qRT-PCR) (Fig. 2h). Moreover, the regulation of RNF40-mediated H2Bub1 on the genes was confirmed by rescue experiments of *Rnf40* in our previous report^12^. Thus, H2Bub1 occupies the transcribed region of cell cycle-related genes and its loss following *Rnf40* inactivation is accompanied by decreased occupancy of TSS-proximal activating histone modifications and RNAPII occupancy and reduced mRNA levels.

### RNF40 regulates cell lineage and pluripotency genes in epigenetic reprogramming

**Fig. 3:**
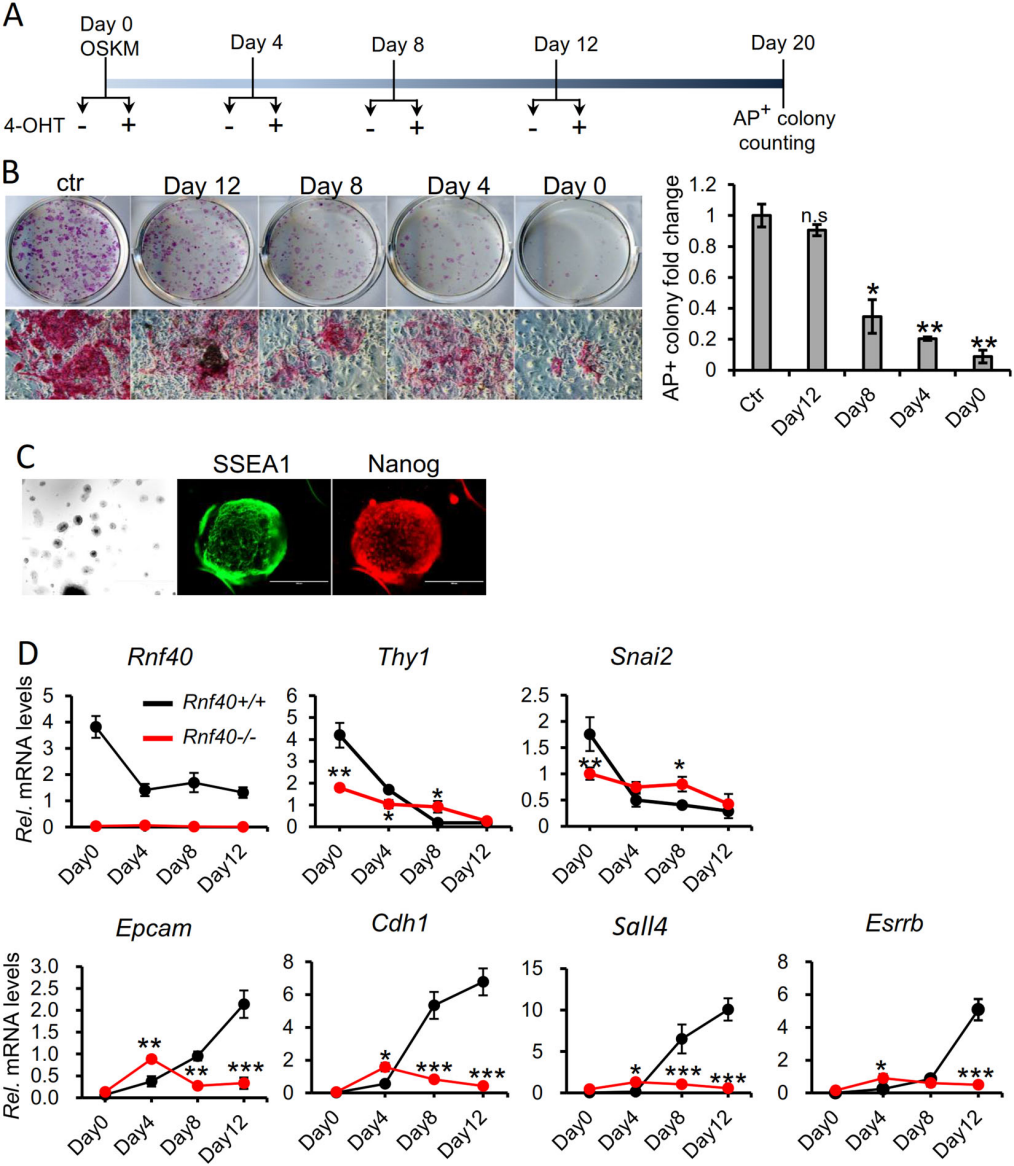
RNF40 is required for the induction of pluripotency gene expression during reprogramming. **a** Experimental scheme of time course analysis of inducing *Rnf40* knockout in homozygous MEFs by adding 4-OHT. iPSC were generated as in Fig. 1a. *Rnf40* was deleted at various time points during reprogramming by adding 4-OHT at day 0, 4, 8, and 12 following OSKM transduction. **b** (Left graph) AP staining of iPSC colonies generated from OSKM-transduced MEFs with *Rnf40* deletion at different times (day 0, 4, 8, 12, or control). The upper images show the AP+ iPSC colonies on the whole wells; the lower images show the single AP+ iPSC colonies at various conditions. Experiments were repeated twice independently. Right graph displays the quantitation of AP+ iPSC colony number under the different conditions. Data ware shown as “AP+ colony fold change”, mean ± SD (*n* = 2 independent experiments). *p < 0.05, **p < 0.001, **p < 0.0001, n.s: *p* > 0.05, calculated with two-tailed unpaired *t*-test. **c** Immunostaining of pluripotency proteins (SSEA1 and Nanog) in iPSC generated using feeder-free N2B27 2i/LIF media. **d** qRT-PCR analysis of time-dependent expression of *Rnf40*, the fibroblast-specific gene (*Thy1*) and pluripotency genes (*n* = 3 independent experiments). Mesenchymal markers (*Cdh2* and *Snai2*); epithelial markers (*Cdh1* and *Epcam*); pluripotency markers (*Sall4* and *Esrrb*). *p < 0.05, **p < 0.001, **p < 0.0001, n.s: *p* > 0.05, calculated with two-tailed unpaired *t*-test.

Somatic cell reprogramming is a highly ordered process, which goes through three phases termed initiation, maturation, and stabilization^43^. We next sought to characterize the functional role of RNF40 in controlling these phases. Thus, *Rnf40* deletion was induced at distinct time points (days 0, 4, 8, and 12) after OSKM transduction and alkaline phosphatase-positive colonies were counted at day 20 (Fig. 3a). Interestingly, the number of AP+ colonies was significantly reduced when *Rnf40* was deleted on days 0, 4, or 8, while there was little or no effect when *Rnf40* was deleted on day 12 (Fig. 3b). Furthermore, the AP+ colony size and AP expression displayed a clear reduction in each knockout condition compared with control (Fig. 3b, lower panel). Thus, these findings indicate that RNF40 is required throughout the entire somatic cell reprogramming process.

Reprogramming of somatic cells to pluripotency is the result of a cascade of transcriptional and epigenetic events^44^. We next investigated the effects of *Rnf40* deletion on different transcriptional events during OSKM-induced reprogramming. Somatic cell reprogramming was performed in serum-free and feeder-free stem cell media containing inhibitor-based 2i/LIF, by which iPSC were formed with high reprogramming efficiency and displayed high expression of the pluripotency markers SSEA1 and Nanog (Fig. 3c). As shown in Fig. 3d, and consistent with previous findings demonstrating increased H2Bub1 levels during differentiation of ESC^10,13^, *Rnf40* expression decreased substantially four days after OSKM transduction and remained constant thereafter. Consistent with previous studies, the MEF-specific gene *Thy1* and the mesenchymal marker *Snai2* were both downregulated during reprogramming, while the epithelial markers *Epcam* and *Cdh1*, as well as the pluripotency markers *Sall4* and *Esrrb*, were continuously upregulated during the course of reprogramming (Fig. 3d). Notably, while *Rnf40* deletion resulted in the downregulation of *Thy1* and *Snai2* at early time points (i.e., day 0 and 4 for *Thy1* and day 0 for *Snai2*), the reprogramming-induced downregulation was partially reversed at day 8 (Fig. 3d, upper panel), suggesting that *Rnf40*-null MEFs are capable of entering a mesenchymal-to-epithelial (MET) program at early stages of reprogramming, but that a full induction of MET requires RNF40 expression. This finding is supported by GSEA of mRNA-seq which demonstrated that *Rnf40*-null MEFs are significantly enriched for MET-associated genes compared with *Rnf40*-deleted MEFs (Fig. 2b and Supplementary Fig. 2B). Consistently, while mesenchymal markers showed an impaired downregulation following *Rnf40* deletion at days 8 and 12, but not at day 4, we also observed an impaired induction of the epithelial markers *Epcam* and *Cdh1* specifically at days 8 and 12, but an increased induction at day 4 (Fig. 3d). A similar pattern of induction was observed for the intermediate and late pluripotent stem cell makers *Sall4* and *Esrrb*^14,45,46^, whose induction was specifically impaired following *Rnf40* deletion (Fig. 3d, lower panel, right). Thus, our findings suggest that deletion of *Rnf40* impairs the activation of pluripotency genes and suppression of tissue-specific genes.

### Deletion of *Rnf40* influences somatic cell reprogramming by modulating H3K4me3/H3K27me3 balance

**Fig. 4:**
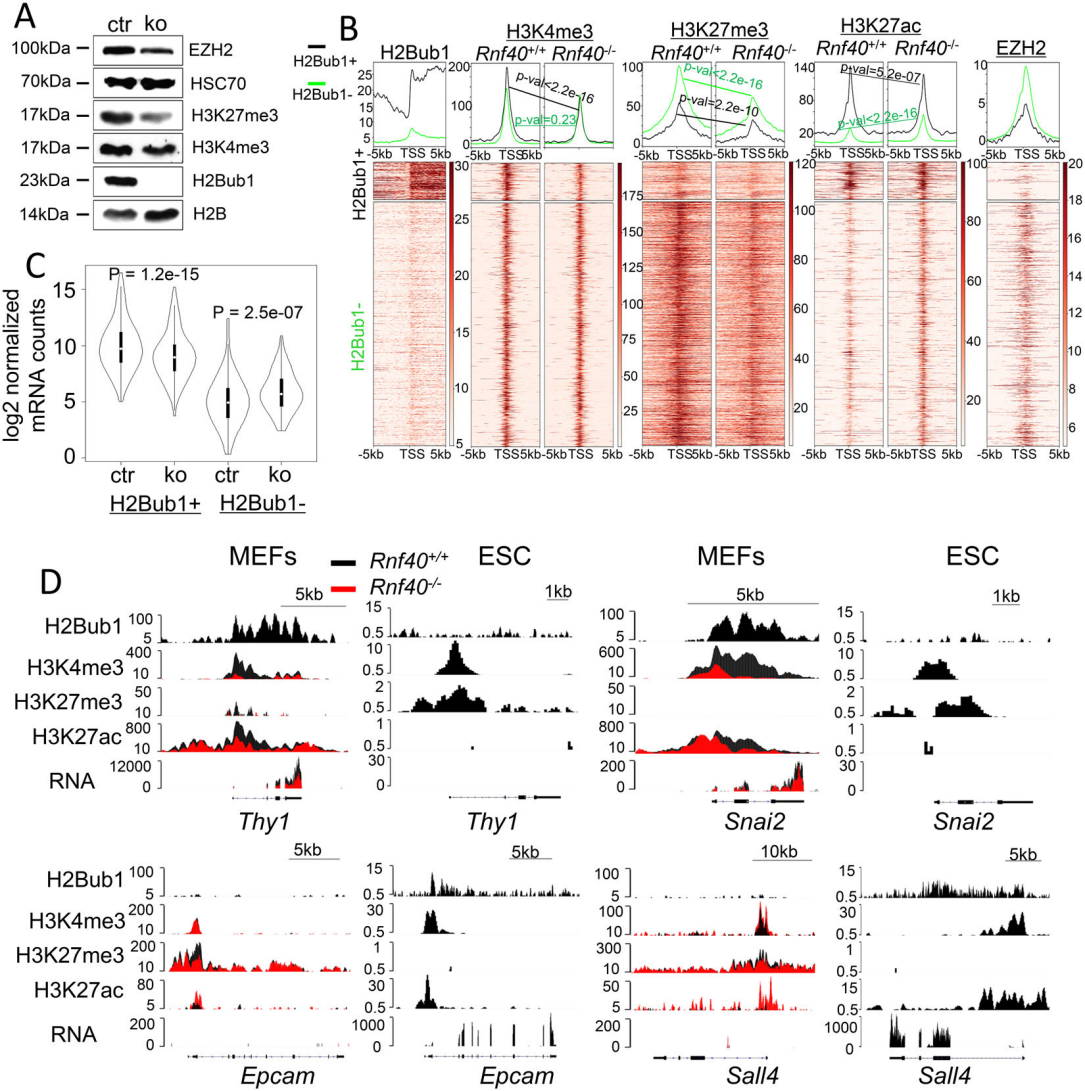
RNF40-mediated H2Bub1 modulates the balance of H3K27me3 and H3K4me3 on bivalent genes regulated during reprogramming. **a** Western blot shows the protein levels of EZH2, H3K27me3, H3K4me3, H2Bub1, HSC70 (loading control), and H2B (loading control) in wild-type (ctr) and *Rnf40*-deleted (ko) MEFs. **b** Aggregate profiles (above) and heatmaps (below) show the occupancy of H2Bub1, H3K4me3, H3K27me3, H3K27ac, and EZH2 surrounding the TSS (±5 kb) on bivalent genes in homozygous MEFs. Genes were clustered by K-means method into H2Bub1-positive (+) genes (up) and H2Bub1-negative (−) genes (down) in response to *Rnf40* deletion. *P*-values were calculated using the paired Wilcoxon–Mann–Whitney test. **c** Boxplots compare the expression levels of H2Bub1-positive and H2Bub1-negative genes between wild-type and *Rnf40*-deleted MEFs. *P*-values were calculated using the unpaired Wilcoxon–Mann–Whitney test. **d** The profiles show the occupancy of H2Bub1, H3K4me3, H3K27ac, and H3K27me3 as well as normalized RNA reads on the *Thy1*, *Snai2*, *Sall4*, and *Epcam* in *Rnf40*^+/+^ (black) and *Rnf40*^−/−^ (red) MEFs. Normal *Rnf40*^+/+^ mouse ESCs are shown for comparison.

The importance of H3K4me3 and H3K27me3 co-occupancy (H3K4me3/H3K27me3) on genes during somatic cell reprogramming to a pluripotent state has been extensively described^14,33,47^. Consistent with our previous studies in which we identified an important role for RNF40-mediated H2B monoubiquitination in controlling the balance between H3K4me3 and H3K27me3^11,12^, we observed a significant decrease in global H3K27me3 levels and a moderate decrease in H3K4me3 following *Rnf40* deletion (Fig. 4a). Based on these findings, we further investigated the importance of RNF40 in controlling H3K4me3/H3K27me3 co-occupied bivalent domains.

We identified 2,041 genes co-occupied by H3K4me3 and H3K27me3 in primary mouse embryonic fibroblasts (Fig. 4b). Interestingly, a subset of H3K4me3/H3K27me3 co-occupied genes, which displayed low levels of transcription, displayed significant levels of H2Bub1. We therefore clustered the bivalent genes and classified them as either H2Bub1-positive (H2Bub1+) or -negative (H2Bub1−) (Fig. 4b). Interestingly, loss of H2Bub1 was accompanied by a moderate decrease in H3K4me3 occupancy in the H2Bub1-positive cluster, in keeping with a genome-wide crosstalk between H2Bub1 and H3K4me3^12^, whereas this effect could not be observed on H2Bub1-negative bivalent genes (Fig. 4b). Consistent with our previous findings showing that RNF40-mediated H2B monoubiquitination controls H3K27me3 levels by maintaining the expression of the methyltransferase EZH2 (Fig. 4a)^12^, H3K27me3 occupancy was significantly decreased both in the H2Bub1-negative and H2Bub1-positive clusters (Fig. 4b). In addition, H3K27ac occupancy was clearly decreased on genes within the H2Bub1-positive cluster, but increased on genes within the H2Bub1+ and H2Bub1− clusters (Fig. 4b). As a result, the effects of H2Bub1 loss on gene expression following *Rnf40* deletion were closely coupled to the changes of these histone modifications, where H2Bub1-positive genes were generally downregulated and H2Bub1-negative genes were generally upregulated (Fig. 4c). Analysis of ChIP-seq profiles confirmed that the epithelial marker genes *Epcam* and the pluripotency gene *Sall4* displayed no significant enrichment for H2Bub1 in MEFs, and exist in a poised bivalent state with H3K4me3 and H3K27me3 co-occupancy in MEFs, but exist in a fully active state displaying both H2Bub1 occupancy and broad H3K4me3, which extends into the transcribed region, in pluripotent stem cells (Fig. 4d and Supplementary Fig. 3)^14,48^. Consistent with the role of RNF40-mediated H2B monoubiquitination in controlling *Ezh2* gene expression and downstream H3K27 methylation^12^, H3K27me3 occupancy displayed a clearly reduction near the TSS of *Sall4* and *Epcam*, which corresponds to the upregulation of those genes following *Rnf40* deletion at the early stage of reprogramming (Figs. 3d and 4d, Supplementary Fig. 3). Moreover, the MEF-specific gene *Thy1* and mesenchymal marker *Snai2* displayed significant H2Bub1 occupancy and broad H3K4me3 domains accompanied by high expression in wild-type MEFs, while these genes are maintained in a poised bivalent state in pluripotent stem cells (Fig. 4d and Supplementary Fig. 3). Consistent with our finding that H2Bub1 facilitates gene expression via controlling H3K4me3 spreading into the gene body and the establishment of broad H3K4me3 domains^12^, loss of H2Bub1 resulted in the narrowing of the H3K4me3 domain and a significant decrease in *Thy1* and *Snai2* expression in the early reprogramming phase (Figs. 3d and 4d). Thus, our findings suggest that RNF40-mediated H2B monoubiquitination plays a central role in controlling the balance between H3K4me3 and H3K27me3 on both cell-lineage and pluripotency genes during differentiation and epigenetic reprogramming, respectively.

## Discussion

**Fig. 5:**
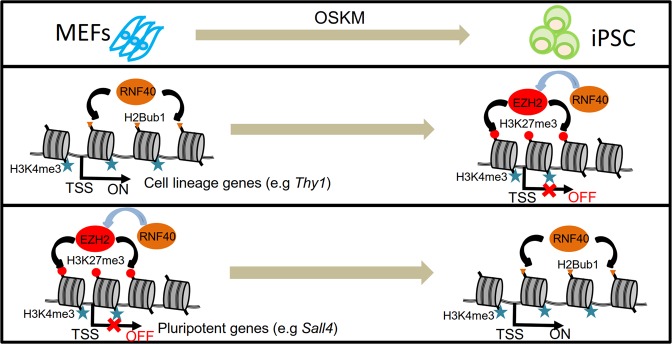
Model depicting how RNF40 regulates fibroblast-specific genes and pluripotency genes during epigenetic reprogramming. The model shows the necessity of RNF40 both in maintaining the expression of cell lineage genes (middle panel) as well as the induction of pluripotency genes (lower panel).

Epigenetic regulators have long been hypothesized to play a central role in the epigenetic resetting of the genome during the reprogramming of somatic cells to pluripotent stem cells^5,7,49,50^. Here we investigated the effect of *Rnf40* deletion and the accompanying loss of H2Bub1 on somatic cell reprogramming induced by the Yamanaka factors. Our work not only establishes an important role of RNF40-mediated H2B monoubiquitination for the expression of central cell cycle regulators, but also uncovers a previously unknown function of H2Bub1 in the transcriptional upregulation of cell proliferation during the earliest stage of somatic cell reprogramming. Furthermore, we show for the first time that H2Bub1 has a dual function in cell fate determination in which it is not only required for full silencing of somatic cell lineage genes but also for the activation of pluripotency genes during cellular reprogramming (Fig. 5). On the one hand, the full silencing of tissue-specific genes requires H2Bub1-mediated activation of the *Ezh2* gene in order to establish bivalency at a select group of promoters (Fig. 5, middle panel). On the other hand, H2Bub1 is also required for the activation of pluripotency genes via the establishment and maintenance of broad H3K4me3 domains (Fig. 5, lower panel). Interestingly, one study identified RNF40 as being a barrier to human iPSC generation^51^. Notably, this study was performed with shRNA-mediated knockdown, rather than complete gene ablation. This finding would be consistent with our previous work as well as the work of Fuchs et al. and Chen et al. showing that H2Bub1 levels are low in stem cells and increase during differentiation^10,11,13^. Thus, it is possible that a reduction of H2Bub1 levels caused by a knockdown of RNF40 may, in fact, promote the induction of pluripotency, while the complete loss of H2Bub1 caused by the genetic deletion of *Rnf40* has a much more severe effect, as shown in our data. In addition, the opposite findings may be also due to the cell type differences. RNF40-mediated H2Bub1 might potentially be involved in different regulatory networks during human and mouse iPSC generation. To address this, we investigated the expression levels of genes identified by Qin et al. to be RNF40-controlled reprogramming barriers. Among these genes *Ptpn11*, *Tmf1*, *Ptprj*, and *Med19* were significantly increased, while *Atf7ip* was decreased in *Rnf40*-deleted MEFs (Supplementary Fig. 4), supporting that there may, indeed, be organism-specific differences in RNF40 and H2Bub1 function during reprogramming.

### Deletion of *Rnf40* impairs early gene activation in reprogramming

A mechanistic explanation for the low efficiency and long latency of somatic cell reprogramming is a stochastic model in which an individual somatic cell is thought to need to overcome various epigenetic barriers to be reset to a pluripotent state^52^. Recently, a subset of privileged fibroblasts was identified, whose cell cycle acceleration reaches a critical threshold, which enables reprogramming to pluripotency to occur in a non-stochastic manner^16^. Indeed, the first transcriptional event in Yamanaka reprogramming is the upregulation of genes that increase cell proliferation^14,33^. Consistent with the observation in other cell systems^21^, a loss of RNF40-mediated H2B monoubiquitination resulted in impaired cell proliferation in MEFs. Furthermore, we observed an enrichment of H2Bub1 on the bodies of cell cycle-related genes activated during the early stages of induced pluripotency. Consistently, the loss of H2Bub1 results in significant downregulation of those genes, indicating that RNF40-mediated H2B monoubiquitination directly participates in the activation and/or maintenance of the expression of these genes. We suggest that the loss of H2Bub1 leads to a significant decrease in the fraction of privileged somatic cells by inhibiting cell proliferation.

While most active genes display a significant occupancy of H2Bub1, until now it was not known that cell cycle-related genes are highly and particularly dependent on H2Bub1. Interestingly, consistent with our previous observation that transcriptional dependency on H2Bub1 occurs primarily on genes displaying low or moderate enrichment of H2Bub1^12^, the early activated cell cycle-related genes in reprogramming display low or moderate levels of H2Bub1. In addition, RNA Polymerase II highly occupies their promoters, with significantly lower levels across the gene body, indicating that the early activated genes maintain a low transcriptional elongation rate. Thus, H2Bub1 may be required to facilitate the transcriptional elongation on these genes. Indeed, many positive transcriptional elongation factors (such as CDK9, BRD4, ELL2, AFF4, and LEO) are able to increase cell proliferation^53–56^. Moreover, an intimate connection between cell cycle and cell fate determination has been shown in a number of cell types^57–59^. While more studies are required to determine the relationship between transcriptional elongation, cell cycle and epigenetic reprogramming, our work provides additional insight that H2Bub1 may be a central mediator of the effects of many of these other proteins, since its activity is also directly linked to their expression and activity^24,60–62^.

### H2Bub1 controls the balance between H3K4me3 and H3K27me3 on tissue-specific and pluripotency genes in reprogramming

Chromatin dynamics play an essential role in controlling cell identity, and changes in activating or repressive histone marks on tissue-specific and pluripotency genes are required for efficient epigenetic reprogramming and cell differentiation^14,33^. In somatic cell reprogramming, the differential expression of bivalent cell lineage genes is directed by the early loss of H3K4me3 and concomitant increase in H3K27me3 on repressed genes^33^. Consistent with the finding that lineage-specific gene expression requires H2Bub1 to establish and maintain broad H3K4me3 domains^12,63^, we observed that loss of H2Bub1 resulted a narrowing of the TSS-associated H3K4me3 peak on the fibroblast-specific gene *Thy1* as well as the mesenchymal gene *Snai2*, thereby resulting in their decreased transcription at the early stage of reprogramming.

During somatic cell reprogramming, lineage-specific genes were repressed, and transitioned from an active state (occupied with active histone markers H3K4me3, H3K27ac, H2Bub1, etc.) to a bivalent state (occupied by both the active histone mark H3K4me3 as well as the repressive histone mark H3K27me3) or to a repressed state (occupied by repressive histone marks such as H3K27me3, H3K9me3, H4K20me3, etc.). In contrast, pluripotency genes were gradually activated and proceeded from a repressed state (occupied by repressive histone marks such as H3K27me3, H3K9me3, H4K20me3, etc.) or a bivalent state (simultaneously occupied by the active histone marks H3K4me3 and the repressive histone mark H3K27me3) to the active state (occupied by H3K4me3, H3K27ac, H2Bub1, etc.)^14^. The important role of active or repressive histone modifications on lineage-specific genes and pluripotent genes might be different during diverse processes in reprogramming. For example, the downregulation of lineage-specific genes mainly resulted from the loss of active histone markers at the early stage. The loss of H2Bub1 results in a further decrease in H3K4me3 and concomitant downregulation of lineage-specific genes at this stage. However, when lineage-specific gene expression is reduced to a threshold (in the bivalent state), the further repression of lineage-specific genes appears to require the additional occupancy of repressive marks like H3K27me3^32^. Therefore, loss of H2Bub1 derepressed lineage-specific genes in the bivalent state indirectly via decreasing the EZH2-mediated H3K27me3 occupancy. In contrast, a derepression does not appear to be sufficient for the induction of pluripotency genes. Instead, the activation of pluripotency genes seems to require an epigenetic threshold, dependent on the occupancy of active histone markers^32^. Thus, while loss of H2Bub1 resulted in the loss of H3K27me3 on these genes, its loss also prevented the activation of pluripotency genes during the later stage. Together, these data strongly support that RNF40-mediated H2B monoubiquitination plays a particularly important role in controlling transcriptional reprogramming in cell state transitions.

The full repression of tissue-specific genes at later stages of reprogramming requires EZH2-catalyzed H3K27me3 to establish promoter bivalency^5,14^. Given the fact that the expression of *Ezh2* is highly dependent on H2Bub1, the loss of H2Bub1 also leads to impaired silencing of tissue-specific genes at the later stages of reprogramming. We further observed that many pluripotency genes displayed significant H2Bub1 occupancy in ESC cells, but are silenced in a bivalent state in somatic cells, indicating that, in addition to its indirect role in maintaining a repressive state (through EZH2), H2Bub1 likely also participates directly in the activation of pluripotency genes during reprogramming. This effect is likely related to the role of H2Bub1 in facilitating the resolution of bivalency^11^. Further studies will be necessary to determine the exact mechanistic role(s) of H2Bub1 in the activation of pluripotency genes during reprogramming.

Together our data provide new insights into the instructive roles of RNF40-directed H2B monoubiquitination in somatic cell reprogramming. H2Bub1 affects somatic cell reprogramming through both cell cycle-dependent and -independent mechanisms. Loss of H2Bub1 impaired somatic cell reprogramming by inhibiting the early activation of genes associated with increased cell proliferation. Moreover, our data support a model in which the full activation of pluripotency genes requires H2Bub1 to promote the spreading of H3K4me3 into the gene body. In addition, given the finding that RNF40 and H2Bub1 directly trigger the expression of the H3K27 methyltransferase *Ezh2*, our data support a model in which full suppression of tissue-specific gene transcription by RNF40-dependent H2B monoubiquitination is mediated through EZH2. Thus these data provide important insight into the multiple mechanisms by which RNF40 functions as a central player during somatic cell reprogramming and confirm the importance of chromatin dynamics in cell fate transitions.

## Supplementary information

Supplementary Figure Legends, Supplementary Tables S1-S3

Supplementary Table S4

Supplementary Figure S1

Supplementary Figure S2

Supplementary Figure S3

Supplementary Figure S4
