# Supplementary material for: The H2B ubiquitin-protein ligase RNF40 is required for somatic cell reprogramming
Source: Cell Death Dis. 2020 Apr 27;11(4):287. doi: 10.1038/s41419-020-2482-4 (PMC7184622; doi:10.1038/s41419-020-2482-4)
Supplement: Supplementary file 1 — Supplementary Figure Legends, Supplementary Tables S1-S3 [file 41419_2020_2482_MOESM1_ESM.docx]

**Supplementary Information**

**Supplementary Figure Legends**

**Supplementary Fig. 1. Induction of *Rnf40* knockout in MEFs. Related to Figure 1** (A) Genotyping of transgenic mice by PCR. (B) PCR for the *Rnf40*-loxP loci following 250 nM of 4-OHT treatment for 24, 48, and 120 h. (C) Western blot shows the protein level of RNF40, ACTB, H2Bub1, and H2B in WT, loxP/wt, and loxP/loxP MEFs with or without 4-OHT treatment. MEFs were cultured with 250 nM of 4-OHT for 120 h to induce *Rnf40* deletion. (D) Immunostaining of pluripotency proteins SSEA1 and Nanog in iPS cell colonies at 12 and 20 d after OSKM transduction of wildtype MEFs.

**Supplementary Fig. 2. Supplementary Figure S2. RNF40 controls early gene expression in reprogramming. Related to Figure 2** (A) Gene Ontology (GO) analysis of significantly upregulated (UP) and downregulated (DOWN) genes at day 1, 3, and 5 after OKSM transduction. Cell cycle or EMT-related gene terms showing p-value < 0.01 have been displayed in the table. (B) GSEA on mRNA-seq data revealed a significant activation of EMT-related genes in *Rnf40*^–/–^ MEFs. NES, normalized enrichment score; FDR, false discovery rate. (C) GSEA on mRNA-seq data revealed a significant decrease in the expression of cell cycle-related genes in *Rnf40*^–/–^ MEFs. (D) The cell cycle pathway. * indicated significantly downregulated genes in *Rnf40* deletion. (E) Heatmap showing the cell cycle-related genes in con (wildtype) and ko (Rnf40 deletion) MEFs. The differential expressed genes at early reprogramming phase were generated by analyze the expression array data (GSE67462). Upregulation of cell cycle-related genes were identified by GO analysis on the differential expressed genes. The effect of Rnf40 deletion on those genes was showing in heatmap. (F) Heatmap showing the EMT-related genes in con (wildtype) and ko (Rnf40 deletion) MEFs. The differential expressed genes at early reprogramming phase were generated by analyze the expression array data (GSE67462). Downregulation of EMT-related genes were identified by GO analysis on the differential expressed genes. The effect of Rnf40 deletion on those genes was showing in heatmap.

**Supplementary Fig. 3. Dynamic changes of active and repressive histone markers on tissue-specific genes and pluripotent genes. Related to Figure 4.** The profiles show the occupancy of H3K4me3, H3K27ac, and H3K27me3 on the *Thy1*, *Snai2*, *Sall4*, and *Epcam* in iPSCs and MEFs distinct time points after OSKM transduction.

**Supplementary Figure S4.** Heatmap showing the gene regulation in con (wildtype) and ko (Rnf40 deletion) MEFs. Those genes were identified by Qin, et al. as a RNF40-regulated barrier in human iPSC generation. * indicated significantly.

**Supplementary Table 1: Antibodies used for western blot**

|  | **Clone** | **Cat.No.** | **WB** | **Source** |
| --- | --- | --- | --- | --- |
| HSC70 | B-6 | sc-7298 | 1:50,000 | Santa Cruz |
| H2B | 53H3 | 2934 | 1:1000 | Cell signaling |
| RNF40 | - | 15621-1-AP | 1:1000 | Acris |
| EZH2 | - | 4905 | 1:1000 | Cell signaling |
| H2Bub1 | 7B4 | - | 1:10 | Hybridoma (Prenzel et al., 2011) |
| H3K27me3 | - | pAb-195-050 | 1:1000 | Diagenode |
| H3K4me3 | - | pAb-003-050 | 1:1000 | Diagenode |

**Supplementary Table 2: Primers for qRT-PCR and ChIP-qPCR**

|  | **Gene** | **Forward sequence** | **Reverse sequence** |
| --- | --- | --- | --- |
| qRT-PCR primers | *36B4* | TTGGCCAATAAGGTGCCAGC | CTCGGGTCCTAGACCAGTGT |
|  | *Ezh2* | TCCATGCAACACCCAACACA | AACTCCTTAGCTCCCTCCAGAT |
|  | *Rnf40* | GCCACACTCCTCATCGTCAA | CTGGGAGAGGAGTCCCATCA |
|  | *Top2a* | GGTTTTACGGAGCCAGTTTTATAGG | TCACGTCAGAGGTTGAGCAC |
|  | *Aurkb* | GCACCTGAAACATCCCAACATCC | CCTTGTAGAGTTCCCCGCGA |
|  | *Bub1* | GTGGTGGAATTGTCACACAAGG | GGGAGCAAGTATTTTGTCCAACAC |
|  | *Cenpa* | CAGGACCAAGAAGGCTTAGGG | AGTGGACTTGGCCCTCTACA |
|  | *Igf1* | ATACCTGCCTGGGTGTCCAA | ACAGCAGGTCAGAGTGGGTA |
|  | *Cdk1* | AAGTGTGGCCAGAAGTCGAG | TCGTCCAGGTTCTTGACGTG |
|  | *Vegfa* | GCAGCGACAAGGCAGACTAT | AACCTCCTCAAACCGTTGGC |
|  | *Plk1* | CGCTGGCGAAAGAAATTCCG | TCCTTTACCCAGAAAGCGCC |
|  | *Thy1* | GTCTTGCTTCTCCCGGTCAG | GGACAAGGTCCCCGTTTCTC |
|  | *Snai2* | CGATGCTATAGGACCGCCG | CAGTTCGCTGTAGTTGGGCT |
|  | *Epcam* | TGGACCTGAGAGTGAACGGA | CGGGTGCCTTTTCATCAACG |
|  | *Cdh1* | AACCCAAGCACGTATCAGGG | GAGTGTTGGGGGCATCATCA |
|  | *Sall4* | ATTACTGGGACATGCGCGTT | GGAAGAGCCCTGGTGACTTG |
|  | *Esrrb* | AATCCTGGTCCTCCCCCTTT | CCCCATGCAAGCTTCGTAGT |
| ChIP-qPCR primers | *Cdk1-promoter* | CAGGAGCTTAGAGGCCGAGT | GAACGTCTACGTGCAATCGGA |
|  | *Cenpa-promoter* | CCAGTGTAGGTAAGCGAGCC | GACCAGGAACCGTCCCTCTA |

**Supplementary Table 3: Publically available datasets**

| Cell type | Data name | Data ID | source |
| --- | --- | --- | --- |
| MEF | H2Bub1 ChIP-seq | GSE72237 | (Xie et al. 2017) |
|  | H3K4me3 ChIP-seq |  | (Xie et al. 2017) |
|  | H3K27me3 ChIP-seq |  | (Xie et al. 2017) |
|  | H3K27ac ChIP-seq |  | (Xie et al. 2017) |
|  | RNA Polymerase II ChIP-seq | GSE61523 | (Wang et al. 2014) |
|  | mRNA-seq | GSE72238 | (Xie et al. 2017) |
|  | EZH2 ChIP-seq | GSM905453 | (Pinter et al. 2012) |
| ESC | H2Bub1 ChIP-seq | GSE76570 | (Lee et al. 2017) |
|  | H3K4me3 ChIP-seq | GSE39513 | (Jia et al. 2012) |
|  | H3K27me3 ChIP-seq |  |  |
|  | mRNA-seq |  |  |
|  | H3K27ac ChIP-seq | GSE42152 | (Banaszynski et al. 2013) |
| iPSC | H3K4me3 ChIP-seq | GSE67520 | (Chen et al. 2016) |
|  | H3K27me3 ChIP-seq |  |  |
|  | H3K27ac ChIP-seq |  |  |
