# Supplementary figures and images for: The H2B ubiquitin-protein ligase RNF40 is required for somatic cell reprogramming
Source: Cell Death Dis. 2020 Apr 27;11(4):287. doi: 10.1038/s41419-020-2482-4 (PMC7184622; doi:10.1038/s41419-020-2482-4)

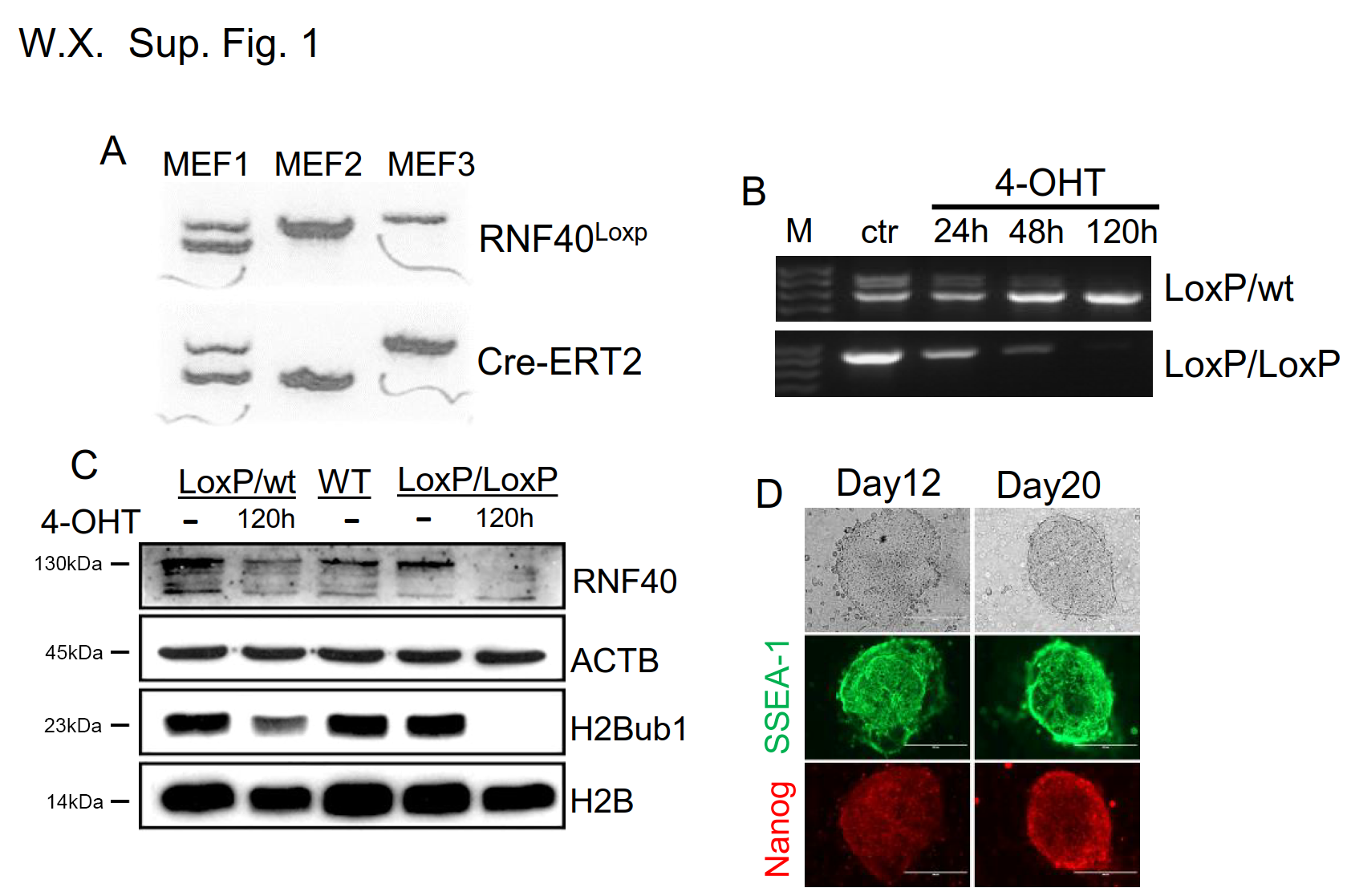

Supplement: Supplementary file 3 — Supplementary Figure S1 [file 41419_2020_2482_MOESM3_ESM.tif]

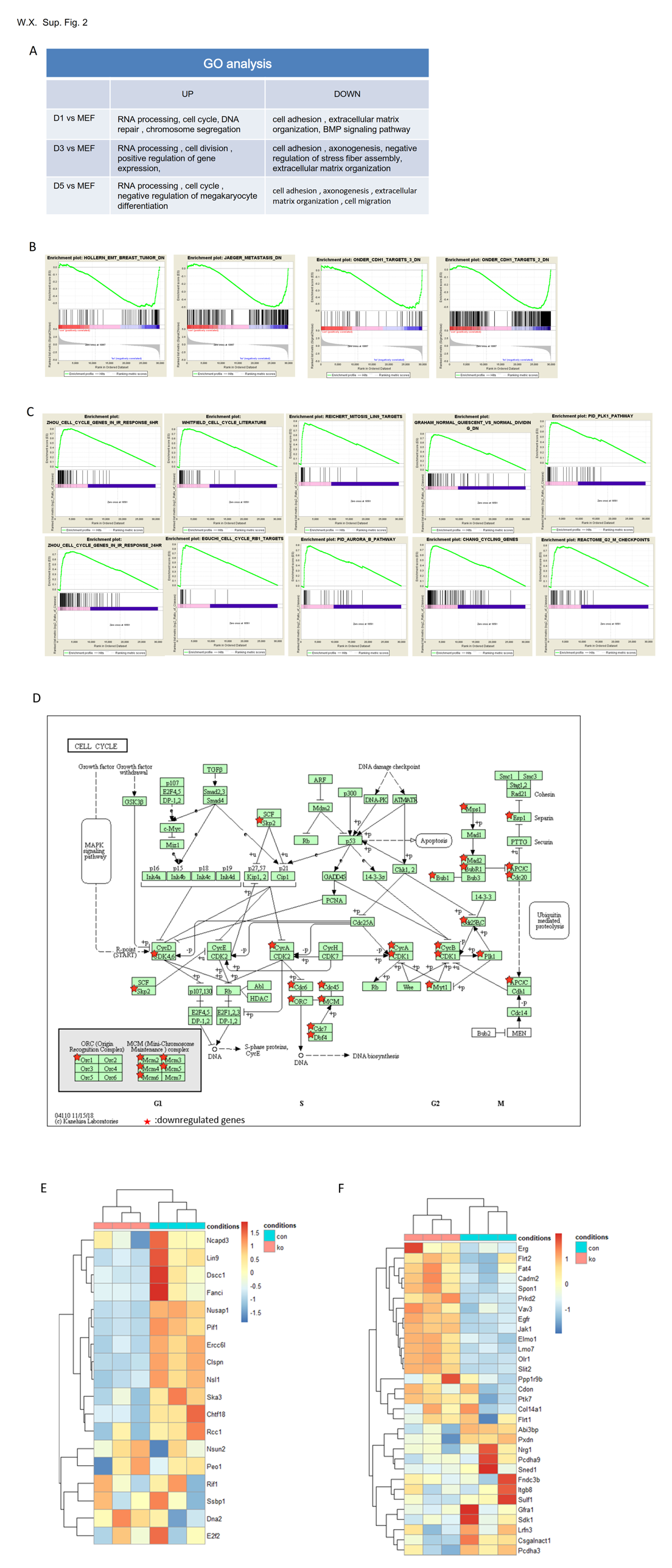

Supplement: Supplementary file 4 — Supplementary Figure S2 [file 41419_2020_2482_MOESM4_ESM.tif]

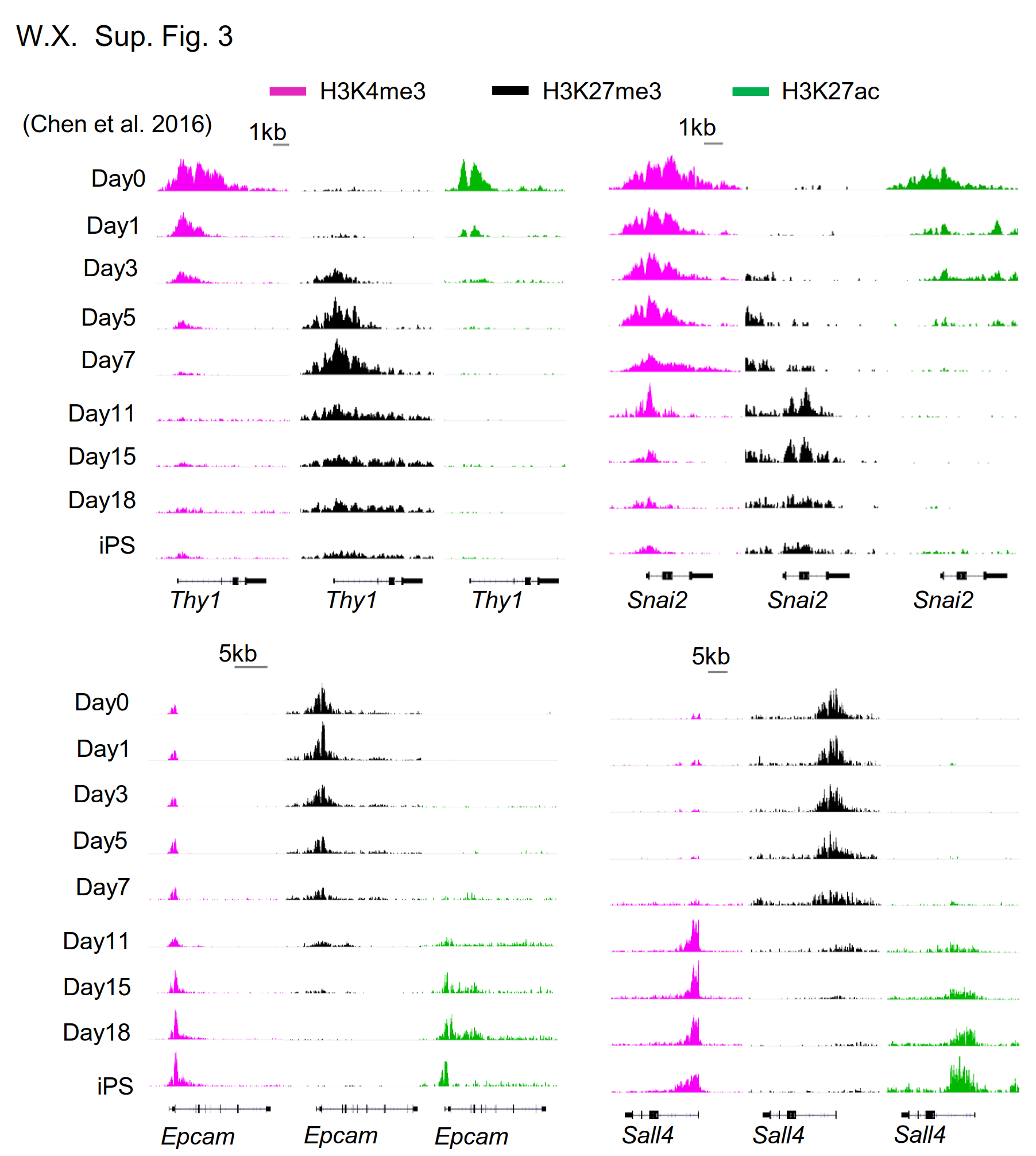

Supplement: Supplementary file 5 — Supplementary Figure S3 [file 41419_2020_2482_MOESM5_ESM.tif]

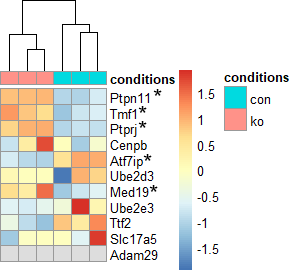

Supplement: Supplementary file 6 — Supplementary Figure S4 [file 41419_2020_2482_MOESM6_ESM.tif]
